# Access to veterinary care in Canada: a cross-sectional survey of animal healthcare organizations and interventions

**DOI:** 10.3389/fvets.2025.1581316

**Published:** 2025-05-30

**Authors:** Quinn Rausch, Maryam Alhamdan, Shane Bateman, Michelle Evason, Valli Fraser-Celin, Courtney Graham, Jamie Saad, Michelle Tuma, Karen Ward, Lauren Van Patter

**Affiliations:** ^1^Department of Clinical Studies, Ontario Veterinary College, University of Guelph, Guelph, ON, Canada; ^2^Department of Animal Biosciences, Ontario Agricultural College, University of Guelph, Guelph, ON, Canada; ^3^Antech/Mars Science & Diagnostics, Fountain Valley, CA, United States; ^4^Veterinarians Without Borders North America, Ottawa, ON, Canada; ^5^SPCA de Montréal, Montreal, QC, Canada; ^6^Toronto Humane Society, Toronto, ON, Canada

**Keywords:** access to care, community and shelter medicine, veterinary desert, underserved community, equity

## Abstract

**Introduction:**

Many Canadians struggle to access healthcare for their animals, but little data is available from the Canadian context on how barriers to care are being addressed, and with what effects.

**Methods:**

The aim of this research was to characterize service providing organizations, barrier mitigation tools, community partnerships, and evaluation metrics used by organizations attempting to increase access to animal healthcare in Canada. In this study, we conducted online data mining and a cross-sectional, mixed-methods organizational survey.

**Results:**

Responses to the survey (*N* = 97) were received from non-profit organizations (52%), for-profit clinics (38%), and several municipal or governmental services (4%) and educational institutes (5%). Commonly reported tools included no cost or low-cost services, pop-up clinics and providing items to assist with pet transportation, with many other tools (payment plans without a credit check, services in multiple languages, availability of assistive technology) being employed by fewer than 20% of responding organizations. Only 38% of organizations used at least one tool from each of the four categories of barriers. Community involvement in programs ranged from simply accessing the service when it was available (outreach) to giving occasional feedback on their experiences (consulting), being employed or volunteering in program provision (collaborating), and community leadership partnering on initiatives (sharing leadership). Program evaluation most often involved quantitative measures of service usage with fewer organizations formally soliciting feedback from the community or looking at long-term health impacts.

**Discussion:**

Responses demonstrate that organizations employ a wide range of tools to mitigate access to veterinary care barriers primarily along financial and geographical lines, and to a lesser extent with tools targeting cultural or disability-related barriers highlighting the importance of building capacity around addressing multiple intersecting barriers. Study findings provide a baseline characterization of current efforts by Canadian organizations to mitigate barriers to accessing animal healthcare.

## 1 Introduction

In Canada, an estimated 60% of individuals/families have at least one companion animal, primarily a dog or a cat (1). A significant percentage of these multi-species families in Canada experience barriers to accessing healthcare for their animals. According to extrapolations from a recent survey, at least 1.62 million families face barriers to accessing preventative care, 1.08 million face barriers to accessing sick care, and 719,000 face barriers to accessing emergency care (2). Additionally, using the Index of Care Accessibility, Neal and colleagues (2024) concluded that almost 4 million households across Canada fell within the lowest accessibility ranking. These numbers are expected to be a significant underestimation because communities most likely to face barriers, such as remote Indigenous Nations, those experiencing disability, homelessness, mental illness, and/or unemployment (3, 4), were not included in the survey. Additionally, the Yukon, Northwest Territories and Nunavut, which are the geographic areas with the most significant barriers to accessing care (5, 6) were not mapped using the Index of Care Accessibility.

Barriers to accessing animal healthcare services are diverse and often very context specific. Experiences of barriers to care have been linked to recent immigrants and young age in Canada (2) and ethnicity, low income, young age, geographic area, and lower education level in the United States (7, 8). Overall, the most prominent barriers cited in access to care literature are affordability of care (2, 3, 9–11) and geographic accessibility of care (4, 7, 8, 10–12). Other notable barriers present in the literature include limited personnel/equipment, available transportation, veterinary-client relationship or communication, client identity, appointment availability, client mental/physical circumstances, government support, cultural or language differences, and client education on animal healthcare (2–4, 7, 8, 10–12).

There are significant animal and human wellbeing threats that arise from limited or no access to animal healthcare. As summarized by Pasteur and colleagues in a recent review (2024), access to animal healthcare improves animal physical health (5, 13–24) as measured by disease prevalence (22, 23), body condition scores (20), and population control through increase in spay and neuter percentages (20). Access to care has also been shown to improve behavioral indicators of animal welfare (14, 17) measured by decreased vocalizations related to stress or nervousness (25), and decreased roaming and barking (26). Access to animal healthcare can also prevent the spread of zoonotic diseases to humans (27) and ensures that the human wellbeing benefits of pet companionship (28, 29) do not become stresses and burdens (30). Veterinary professionals are also often a trusted source for animal welfare information and heavily relied upon by animal caretakers (31–34), and lack of access can impede the sharing of best practices in animal care potentially leading to welfare issues from an animal care perspective (11).

In response to the substantial need for more accessible animal healthcare services, many grassroots organizations and multinational corporations have developed programs, employed a range of tools, or taken steps to mitigate barriers with the hopes of improving accessibility locally or for a particular underserved population (35). A notable challenge is that the success of accessible veterinary care programs might vary depending on the underserved community, their animal population and relationship with animals, geographic location, beliefs, barriers, needs, expectations, and priorities (35). Throughout the process of increasing access, it would be helpful for organizations to: (1) define and be able to measure what program success would look like [i.e., what does “access to health care” entail (11, 36)]; (2) determine where to focus their efforts [i.e., locating “veterinary care deserts” (37) or “underserved communities” (38)]; and (3) partner with their target community to understand community-specific barriers and determine tools that would most effectively and appropriately mitigate them. Unfortunately, this might not always be possible for organizations due to lack of resources, emergent situations, or lack of expertise.

To improve access to animal healthcare in Canada and support organizations working in this space, we need to better understand the current organizational landscape: How are organizations across the country attempting to address barriers to accessing animal healthcare in underserved communities? What strategies are being implemented, where, and with which communities? And how are program outcomes measured and evaluated? This paper provides baseline data to answer these questions, drawing on the findings of online data mining and a cross-sectional, mixed-methods organizational survey conducted in 2023. We report how access to veterinary care is being addressed by organizations working with diverse communities across Canada and briefly characterize the services, barrier mitigation tools, community partnerships, and evaluation metrics being used by organizations attempting to increase access to animal healthcare in Canada.

## 2 Materials and methods

All procedures in this study were reviewed and approved by the University of Guelph.

Research Ethics board for compliance with federal and provincial guidelines for research involving human participants (REB #23–07-031).

### 2.1 Data mining

Online data mining was conducted between May and August of 2023 using Google Chrome to search for and make a database of animal healthcare service providing organizations who were advertising efforts to increase financial, geographical, cultural, or physical accessibility of services. Search strings included all combinations of a region (Canada OR individual provinces OR individual cities in the 100 most populated cities according to the 2021 Canadian Census), a barrier mitigation strategy (“low-cost” OR “low cost” OR “low price” OR free OR sponsored OR subsidized OR not-for-profit OR rural OR Indigenous OR “First Nation” OR northern OR mobile OR telehealth OR virtual OR accessible OR “physically accessible” OR “community-based”) and an animal health service (“animal shelter” OR shelter OR humane society OR SPCA OR vet* clinic OR vet* services OR vet* medicine OR vet* program OR vet* treatment OR vaccination OR “spay and neuter” OR “trap neuter release” OR TNR OR animal healthcare). To be included in the database, the organization had to be located and provide services within Canada, have an email address or phone number to contact, and advertise a service or tool that increased geographical, financial, cultural, and/or physical accessibility of animal healthcare services.

### 2.2 Survey development

The survey consisted of 20 questions relating to organizational interventions aimed at reducing barriers to accessing animal healthcare, what tools and methods were used, which communities were targeted, and any measures of program impact that were collected. The survey took approximately 15–20 min to complete. There were single- and multiple-choice and open-ended questions with a textbox for the participant to provide a written answer. Many of the single- and multiple-choice questions had an ‘Other’ option with a textbox to add additional answers. The survey included four sections: (1) consent (information letter and one question collecting informed participant consent), (2) organizational information (four questions relating to organization type), (3) access to care information (11 questions relating to organizational priorities, perspectives, and services offered), and (4) next steps (four questions related to continued participation in other research, contact information, and any additional participant comments; see Supplementary materials for the full survey).

All questions were hypothesis driven and based on current literature on veterinary care deserts (37–41) and barriers and access to animal (4, 8, 10, 12, 42–50) and human (36, 51–55) healthcare in North America. The questionnaire was piloted and reviewed by 7 prominent researchers and practitioners in the access to veterinary care space across Canada and suggestions were incorporated prior to the launch of the survey.

### 2.3 Survey participant recruitment

The survey was open from December 5, 2023, to February 20, 2024. Participation was restricted to individuals 18 years of age or older with the authority to provide details on behalf of an organization offering services that address barriers to accessing animal healthcare in Canada. The online survey hosted through Qualtrics® survey software (56) so internet access was required for participation. The survey and recruitment material were offered and distributed in both English and French. The data mining portion of this project served as the initial recruitment list for the survey distribution with recruitment information sent by email to each organization individually with a follow up reminder sent 1 month after initial contact. If the organization’s email could not be found, the organization was contacted by phone. Snowball sampling techniques were then used to promote the survey further on social media and through larger organizational and regulatory bodies. This convenience sampling method relies on referral from participants and is typically utilized to reach groups of people that are otherwise difficult to access (57, 58).

Initial online advertisements, including the study description and a link to the survey, were posted on Facebook, Instagram, X/Twitter, and LinkedIn. Organizational and regulatory bodies including the Canadian Veterinary Medical Association and each provincial Veterinary Medical Association, Humane Canada, and each provincial shelter association, Canadian Animal Shelter and Community Medicine Association, PetHelpFinder, PetSmart Charities of Canada, the Canadian Kennel Club, International Fund for Animal Welfare, Canadian Companion Animal Health Surveillance Network, and each of the five Canadian veterinary schools were sent recruitment information and asked to distribute it among their members and contacts. Large pet related corporations including Communivet, Zoetis, Elanco, BI, IDEXX, Antech, True North, AHL, PDS, Hill’s, Royal Canin, Purina, and the Veterinary Practice magazine were also sent recruitment information and asked to advertise or distribute the survey to Canadian contacts.

All recruitment material encouraged sharing the survey with acquaintances or contacts who might meet the inclusion criteria. Participation in this research was voluntary, and consent was provided by respondents online through submission of the survey. No incentives were offered for completion of the survey.

### 2.4 Data management

The raw data were downloaded from Qualtrics® survey software and initially cleaned and reviewed in Microsoft® Excel. Any responses that met the following exclusion criteria were removed: [1] answered less than 95% of the survey (*N* = 38), [2] were likely to be a bot response (*N* = 0 based on criteria from (59)), [3] were answered from outside of Canada (*N* = 2), or [4] were duplicates from the same organization (the most complete response was kept, *N* = 3).

### 2.5 Quantitative analysis

Descriptive statistics were generated about participants including frequency and measures of central tendency for organizational type, location, goals, priorities, communities and species served and services offered using STATA statistical software (60).

### 2.6 Qualitative analysis

The open-ended question responses were analyzed using qualitative content analysis with an inductive approach (61). This grounded approach avoids initial researcher bias and allows codes to emerge naturally which encourages exploration of unanticipated insights (61–63). First, all responses were read in full by the lead author (QR) and a codebook was subsequently developed for each qualitative question. Second, all responses for each question were coded using the developed codebook for that question using NVivo 14 qualitative data analysis software (64). Entire responses or sections of responses could be coded under multiple codes. Third, coding was assessed for reliability by comparing coding agreement within NVivo (65) between the first (QR) and last (LVP) authors who both coded 100% of responses. Inter-coder reliability was conducted and yielded agreement of 94%. Any discrepancies were discussed until consensus was reached.

## 3 Results

### 3.1 Data mining

**Figure 1:**
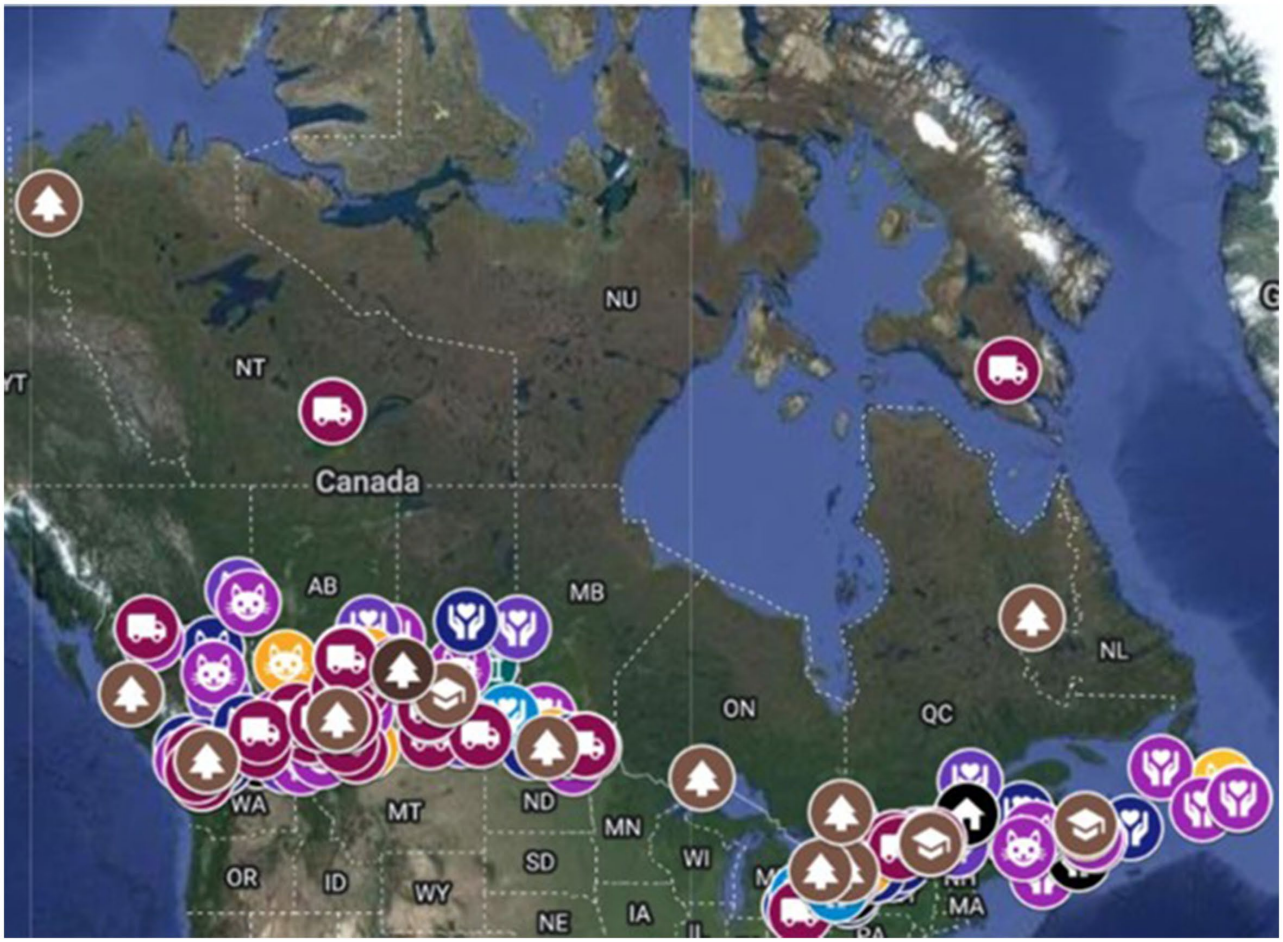
Map showing the geographic distribution of organizations identified offering financially or geographically accessible animal healthcare services in Canada during online data mining from May to August 2023.

The database (*N* = 504) generated from online mining contained primarily for-profit clinics (54%) and non-profit organizations (39%) with some municipal or governmental services (2%) and educational institutions (<1%). Geographic accessibility barriers were predominantly mitigated through mobile private clinics (30%), telemedicine or telehealth appointment options (8%), or pop-up clinics in underserved areas (3%). Financial accessibility was mitigated through free (1%) or reduced cost (10%) spay and neuter services often accompanied by one or more other services like vaccinations or deworming. There were a few complete service clinics that reported being low (2%) or no (<1%) cost and some organizations (1%) that offered pop-up clinics for unhoused populations in large urban centers at no cost. This database will soon become part of an interactive map of organizations in Canada to help pet caretakers find accessible care locally and support organizational collaboration (See Figure 1 for the geographic distribution of organizations).

### 3.2 Quantitative survey analysis

Survey participants (*N* = 97) were most commonly non-profit organizations (52%) or for-profit clinics (38%) with a few municipal or governmental services (4%) and educational institutes (5%; see Table 1 organizational descriptives). Organizations primarily offered services in Ontario (45%), British Columbia (16%), Alberta (14%), and Quebec (12%) with less than 10% in any other province and territory. Organizations primarily offered services for cats (91%) and dogs (89%) with fewer organizations offering services for other small animals (e.g., rabbits, guinea pigs; 49%), exotic animals (e.g., reptiles; 27%), and large animals (e.g., cows, horses; 12%). Participants reported that services were provided by primarily veterinarians (90%) and veterinary technicians (74%) with some organizations allowing other employees or volunteers (47%) to provide services. When organizations were asked to rank their organization’s priorities relative to one another, on average, ‘improving animal welfare’ was the top priority followed by: ‘helping people with pets’, ‘controlling animal populations’, ‘preventing zoonotic disease’, and finally ‘promoting One Health’. In open-ended textboxes asking about other organizational goals not listed above, participants noted: providing education (e.g., student training, humane education, knowledge sharing), improving community safety, rehoming unwanted animals, generating profit, and assisting animal rescue organizations. When asked about which underserved communities they worked with, the most common answer from participant organizations was low-income (66%), followed by Indigenous (51%), rural (47%), unhoused (45%) and northern (31%). Organizations worked on average with between two and three different underserved communities.

**Table 1:** General descriptions, frequencies and measures of central tendency of organizational characteristics collected from 97 animal healthcare providing organizations in Canada who completed the organizational survey between December 5, 2023-February 20, 2024.

Variable	Category	Number (percent) OR mean, median (standard deviation)
Organization purpose	Not for profit organization (NP)	50 (52%)
	For profit clinic (FP)	37 (38%)
	Educational institute (ED)	5 (5%)
	Municipal or government service (MG)	4 (4%)
Organization serving each province	Ontario	44 (45%)
	British Columbia	16 (16%)
	Alberta	14 (14%)
	Quebec	12 (12%)
	Nunavut	8 (8%)
	Saskatchewan	6 (6%)
	Yukon	5 (5%)
	Manitoba	4 (4%)
	Northwest Territories	3 (3%)
	Newfoundland and Labrador	3 (3%)
	New Brunswick	3 (3%)
	Nova Scotia	3 (3%)
	Prince Edward Island	1 (1%)
Number of provinces served by an organization		1.38, 1 (1.5)
Underserved communities targeted	Low-income	64 (66%)
	Indigenous	49 (51%)
	Rural	46 (47%)
	Unhoused	44 (45%)
	Northern	30 (31%)
	Other	6 (6%)
Number of communities served by an organization		2.36, 2 (1.58)
Organizational goal priorities* *Relative importance rank 1 (least) - 6 (most)	Improve animal welfare	4.45, 5 (1.44)
	Help people with pets	4.24, 4 (1.46)
	Control animal populations	3.95, 4 (1.51)
	Prevent zoonotic disease	3.29, 3 (1.31)
	Promote OneHealth	3.21, 3 (1.35)
	Other	1.88, 1 (1.79)
Care providers	Veterinarians	86 (90%)
	Veterinary technicians	72 (74%)
	Other employees/volunteers	46 (47%)
Species served	Cats	88 (91%)
	Dogs	86 (89%)
	Small animals	48 (49%)
	Exotic animals	26 (27%)
	Large or agricultural animals	12 (12%)

The survey participants were asked which tools they use to mitigate affordability, accessibility and cultural or disability related barriers to accessing animal healthcare (see Table 2 for descriptions and frequencies of tools used). In total, only 38% of organizations used at least one tool from each of the four categories of barriers. Affordability barriers were mitigated with an average of 2.26 tools by 93% of organizations. Free (43%) and low-cost services (51%) were the most common tools. Offering funding (37%) and the use of spectrum of care (36%), offering bundled services (e.g., puppy or kitten packages, free services bundled with adoption or purchase; 20%) or payment plans with (13%) or without (15%) credit check requirements were also used by many organizations. In open-ended textboxes asking about other financial accessibility tools, participants noted using: pay what you can, unofficial payment plans, free long-term veterinary support beyond preventative care, offering discounts for particular groups (e.g., seniors, those on government assistance, shelters), free disease control programs or education, accepting volunteer donations to subsidize care for low-income clients, offering vouchers for a service, third party payment plans, reduction in certain costs when bills are high, offering discounts or free services at the veterinarian’s discretion, and staff getting a certain dollar amount per year to use for free services.

**Table 2:** Descriptions, frequencies and measures of central tendency for tools used to mitigate financial, geographic, cultural and disability related barriers to access animal healthcare by 97 animal healthcare providing organizations in Canada who completed the organizational survey between December 5, 2023-February 20, 2024.

Variable	Category	Number (percent) OR mean, median (standard deviation)
Tools used to mitigate financial barriers to accessing animal healthcare	Free or no-cost animal healthcare services	42 (43%)
	Low-cost* or subsidized prices for animal healthcare services *below the average cost of similar services in the community	49 (51%)
	Funding available to assist pet care needs (e.g., grant funds, vouchers)	36 (37%)
	Spectrum of care approach to treatment options	35 (36%)
	Bundled services at a reduced cost	19 (20%)
	Payment plans for animal healthcare services that **do not** require credit checks	15 (15%)
	Payment plans animal healthcare services that **do** require credit checks	13 (13%)
	Other	11 (11%)
	Any of the above	90 (93%)
Number of tools used to mitigate financial barriers by an organization		2.26, 2 (1.45)
Tools used to mitigate geographic barriers to accessing animal healthcare	Provision of items to assist with pet transportation (e.g., carriers)	46 (49%)
	Pop-up or periodic clinics in a different place than the ‘brick and mortar’ address	39 (41%)
	Mobile unit of the clinic	35 (36%)
	Telehealth services	32 (33%)
	Transportation programs or assistance to the clinic	32 (33%)
	Extended hours of operation (outside of 9 am-5 pm Monday–Friday)	30 (31%)
	Other	10 (10%)
	Primary clinic location in a rural, remote or Indigenous community	7 (7%)
	Any of the above	89 (92%)
Number of tools used to mitigate geographic barriers by an organization		2.38, 2 (1.50)
Tools used to mitigate cultural barriers to accessing animal healthcare	Staff cultural safety education or training	40 (41%)
	Diverse staff (specifically equity seeking groups. and/or diversity reflective of your clientele)	28 (29%)
	Offer services in languages other than the dominant language (English or French)	18 (19%)
	Other	6 (6%)
	Any of the above	59 (61%)
Number of tools used to mitigate cultural barriers by an organization		0.95, 1 (0.93)
Tools used to mitigate disability related barriers to accessing animal healthcare	Staff education/training on equity and inclusion of clients with disabilities	39 (40%)
	Physically accessible clinic	39 (40%)
	Assistive technology* *Any technological modality used to assist a person with a disability	4 (4%)
	American Sign Language interpretation	1 (1%)
	Other	10 (10%)
	Any of the above	59 (61%)
Number of tools used to mitigate disability related barriers by an organization		0.96, 1 (0.95)

Geographic accessibility tools were used by 92% of organizations with an average of 2.38 tools used per organization. The most prominent tools employed by organizations were the provision of items to assist with pet transportation (49%), pop-up or periodic clinics in an underserved area (41%), mobile units of the clinic (36%), telehealth services (33%), transportation programs (33%), and having a primary clinic location in a geographically underserved area (7%). In open-ended textboxes asking about other tools to mitigate geographic barriers, participants noted: partnering with external mobile services, facilitating client communication with one another to coordinate a communal vet visit, personal use of staff vehicles for occasional mobile services (e.g., euthanasia, pet pick-up), providing funding for travel, training community/lay vaccinators, removing animals from a community for services and returning them, dropping off medicine or food to clients, helping clients locate a closer service, telehealth consultations between organizations to reduce client travel, and setting up emergency medication pharmacy in a veterinary desert.

Cultural barriers were mitigated by 61% of organizations with the use of 0.95 tools on average used per organization. Tools included staff cultural safety training (41%), having a diverse staff (i.e., equity seeking groups and/or diversity reflective of clientele; 29%), and offering services in multiple languages other than the dominant language (19%). In open-ended textboxes asking about other tools used to mitigate cultural barriers, participants noted: access to a translator when needed, trauma-informed care training, partnering with community leadership and following cultural protocols, focusing on community-led solutions, seeking information from community leadership about needs, employing a social worker, and offering outreach services targeted at equity seeking groups.

Disability-related barriers were mitigated by 61% of organizations with an average of 0.96 tools on average used per organization. The most common tools used were staff training on equity and inclusion of clients with disabilities (40%), having a physically accessible clinic (40%) while a few organizations reported having assistive technology available (4%), and having an American Sign Language interpreter on staff (1%). In open-ended textboxes asking about other tools used to mitigate disability-related barriers, participants noted: offering various formats for filling out paperwork and consents, email correspondence, using trauma-informed and/or neurodiversity aware approaches, taking into consideration disability-related costs when measuring low-income qualification, mobile and in-home services, transportation assistance, meeting clients at vehicles, and use of Google Translate.

### 3.3 Qualitative survey analysis

Content coding was completed for questions 11 (barriers to care), 15 (level of community involvement) and 14 (program impact and evaluation), which are each reported separately below (see Supplementary materials for the full survey). All participant answers quoted are reported exactly as written in the survey with their participant ID directly after (FP = for-profit organization, NP = non-profit organization, MG = municipal or governmental organization, ED = educational institute). When slight clarity, spelling correction, or context was required, edits were added in square brackets.

#### 3.3.1 Barriers to care

Perceived barriers to care fell into five general codes: care affordability, care accessibility, care availability, client situation, and client-service provider relationship (see Table 3 for Codebook with definitions and frequencies). In terms of care ‘**Affordability**’, many participants noted cost of care as a barrier (*n* = 41): “*Rising cost of living and veterinary care*” (FP4); “*difficulty affording the cost of veterinary care*” (MG1); “*Pricing of larger corporate clinics*” (NP24). Several specified challenges related to client disposable income (*n* = 19): “*Finances of pet owners*” (NP24); “*Available money from clients*” (FP23); “*lack of financial resources*” (FP35). Others noted the financial challenges facing organizations that provide access to veterinary care (*n* = 3): “*Limited funds as a non-profit makes it difficult for those tough cases*” (NP8); “*Funding -- our organization is supported by grants and community donations*” (NP45). Respondents also made links to broader systematic financial considerations (*n* = 3): “*Poverty*” (NP7); “*costs of living cause financial barriers*” (FP26); “*the failing economy*” (NP29).

**Table 3:** Qualitative codebook for barriers to animal healthcare including codes, sub-codes, definitions and frequencies reported in qualitative responses by 97 animal healthcare providing organizations in Canada who completed the organizational survey between December 5, 2023-February 20, 2024.

Codes and sub-codes	Definition	# of references
**Affordability**: Barriers related to the affordability of care including financial state and disposable income of clients, cost of care and systemic financial barriers like economic state or cost of living		
Cost of care	Barriers related to the cost of the service including staff salaries, materials, and overall service costs.	41
Client disposable income	Barriers related to the clients’ finances, financial status or disposable income that can be put toward services.	19
Low-cost services	Barriers related to knowledge and availability of low-cost or no-cost clinics.	3
Organizational funding	Barriers related to lack of organizational funding to provide subsidized care.	3
Systemic financial	Barriers related to systemic financial systems including the state of the economy and cost of living.	3
**Accessibility**: Barriers related to the geographic location of the service, client or patient including distance from care, being in a veterinary desert, accessing transportation to the service		
Distance from care	Barriers related to a client’s geographical distance from available services including complete lack of local care.	16
Veterinary desert	Barriers related to complete lack of services or a specific type of service in the local area.	13
Transportation	Barriers related to client and patient access to transportation to the service	18
**Availability**: Barriers related to the quantity or type of services available in an area unrelated to geography including access to appointments or veterinarians taking new clients, species, regulatory and staff shortage issues or quality of care		
Appointments	Barriers related to the availability of service appointments within the timeframe needed including hours of operation, lack of appointments, veterinary space for new clients.	15
Care quality	Barriers related to accessing quality care including handling style, wellness, or preventative care.	2
Regulatory	Barriers related to standards and regulations from veterinary or shelter regulatory bodies or governmental bodies.	5
Species	Barriers related to the species of animal requiring care.	1
Service provider shortage	Barriers related to organizational ability to staff volunteers, veterinary technicians, and veterinarians adequately, number of staff trained in rural, shelter or community medicine or general workforce shortages.	23
Number of animals	Barriers related to the number of animals needing care, size of the population or availability of pets to potential caretakers.	5
**Client situation**: Barriers related to the client’s situation including their health, abilities, knowledge, logistics or situation excluding finances		
Client health and dis/abilities	Barriers related to client mental or physical health or mental and physical dis/abilities.	9
Client knowledge	Barriers related to client level of knowledge, education and information about pet care, value of veterinary care or availability of services.	18
Logistic or situational	Barriers related to the client’s personal situation or resources.	4
**Client-service provider relationship**: Barriers related to the client service-provider relationship including trust of and previous experiences with service providers, cultural differences and bias and service provider knowledge and perspectives on access to care and pet guardianship		
Cultural difference and bias	Barriers related to cultural differences between clients and veterinary professionals including language, religion, cultural perspectives on veterinary medicine or consequences of systemic oppression related to culture or race including colonialism, racism, or discrimination.	13
Trust of service providers	Barriers related to client trust or lack of trust of service providers or previous experiences with a service provider.	7
Service provider knowledge or perspectives	Barriers related to veterinary professionals’ knowledge of access to care, shelter and community medicine or perspectives on access to care, spectrum of care or pet guardianship.	4

In terms of ‘**Accessibility’**, participants discussed barriers related to distance from care (*n* = 16): “*Physical location of where people live, for example, in a very remote location where the closest vet is hours away*” (FP27); “*veterinary clinics are a plane ride [or] 6 + hour drive away*” (NP48); “*location/geographical distance from a veterinarian (so need for transportation and often staying in area to have pet cared for)*” (NP47). Others discussed gaps in veterinary services, or veterinary ‘deserts’ (*n* = 13): “*veterinary deserts*” (NP1); “*no full-time vet care available*” (ED1); “*Lack of services (no emergency services in [location] and only one in all of [location] region that sometimes can be at capacity)*” (NP34). Finally, transportation barriers were frequently noted (*n* = 18): “*Access to transportation*” (NP6); “*Lack of transportation*” (NP34); “*Unable to get to clinics, especially seniors*” (FP29).

Participants also noted barriers in terms of veterinary care ‘**Availability**’, including lack of available appointments (*n* = 15): “*Lack of veterinary clinics accepting patients (availability of for-profit clinic spaces)*” (NP24); “*available clinics wanting to work with rescues*” (NP5); “*appointment accessibility in a reasonable timeframe*” (NP36). Relatedly, many respondents noted the challenges associated with the service provider shortage (*n* = 23): “*The veterinary shortage*” (NP9); “*Trained veterinary staff interested and available to work in shelter medicine*” (NP14); “*Veterinary workforce shortage*” (MG2). Respondents also noted that the high number of animals needing care impacted care availability (*n* = 5): “*Number of animals needing care (due to overpopulation)*” (NP45); “*Free pets are readily available*” (NP15); “*number of pets*” (FP5). Others noted the impacts of regulatory barriers (*n* = 5): “*Regulatory body restrictions*” (NP14); “*current definition of acceptable standard of care in veterinary medicine is too high*” (NP14); “*the regulations of the college that create barriers to helping the general population who cannot afford the cost of care and the limitations of licensing to help organizations like shelters and not for profits who provide care to the forgotten individuals but limit our ability to reach them and provide service*” (NP20).

Responses related to ‘**Client situation**’ acknowledged barriers related to client health and dis/abilities (*n* = 9): “*those in more urban areas who have disabilities (physical or psychological) that make it impossible to get to a local vet*” (NP13); “*mobile disabilities*” (FP7); “*mental health barriers*” (MG4). Client knowledge, both of animal health needs and of service availability, was also noted as a reason some may not access veterinary care (*n* = 18): “*Lack of knowledge or education regarding necessary pet care*” (NP15); “*Education (lack of fluency in health-related needs for animals)*” (NP41); “*Many people encountering this issue do not know where to go for help*” (NP19). Finally, other logistical or situational barriers were noted (*n* = 4): “*Logistical*” (NP30); “*time, effort*” (MG4); “*those in poverty, homelessness, mental turmoil, fleeing violence or emergency situations*” (NP26).

The most commonly noted barrier related to ‘**Client-service provider relationship**’ was cultural differences and bias (*n* = 13). Most responses were very general, stating simply “*cultural barriers*” (NP48), “*cultural acceptability of services*” (ED4), or “*Language/cultural*” (NP30). Several focused on dynamics of service providers in particular, for instance: “*fear of being judged*” (NP1); “*[racism] and discriminatory practises in certain clinics*” (ED5). Others focused more broadly on historical and systemic issues, specifically in the context of access to care barriers faced by Indigenous communities: “*cultural barriers (historic colonialization, racism)*” (ED1); “*Distrust of non indigenous [sic] peoples with the ability to help these communities. Social and racial and financial barriers*” (NP44). One respondent provided a more in depth analysis: “*lack of trust with healthcare providers/animal welfare org[anization]s due in some cases to historical trauma and discrimination or due to practitioner judgement when the owner cannot afford care, culture/linguistic/religious barriers*” (NP43). Respondents also noted barriers related to trust of service providers and previous negative experiences (*n* = 7): “*distrust of medical profession (seeing animal welfare agencies as punitive and worried they will lose their animal);* “*trust in veterinary service providers*” (ED4); “*Past negative experiences in health and veterinary care*” (NP7). Finally, participants also noted that service provider knowledge or perspectives can contribute to barriers (*n* = 4): “*Knowledge of spectrum of care opportunities and organizations involved in spectrum of care*” (NP7); “*lack of practitioner knowledge about shelter medicine*” (NP43).

#### 3.3.2 Level of community involvement

Organizations reported varying levels of community partnership when qualitative responses about community involvement in the program were placed on the following spectrum of participation delineated in the ‘Principles of Veterinary Community Engagement**’** (66): outreach, consulting, involving, collaborating, and sharing leadership (see Table 4 for Codebook with definitions and frequencies). Aligning with an ‘**Outreach’** model, organizations determined community needs on behalf of the communities they work with (*n* = 1), provided information in a unidirectional manner (*n* = 3), and/or simply noted that the community uses their services (*n* = 12): “*We observed that they needed help and started the program ourselves*” (NP39); “*These communities are involved as service recipients*” (NP39).

**Table 4:** Qualitative codebook for level of community involvement in service provision including codes, sub-codes, definitions and frequencies reported in qualitative responses by 97 animal healthcare providing organizations in Canada who completed the organizational survey between December 5, 2023-February 20, 2024.

Code	Definition	# of references
Outreach: some community involvement; unidirectional flow of information/services		
Information sharing	The program provides information to the community about animal care, veterinary medicine or the program itself.	3
Using or offering services	Community use of services offered by the organization	12
Community needs determined by organization	The needs of the community are determined by the organization through observation, assumption or without community engagement.	1
Consult: bidirectional flow of communication, information sharing		
Relevant party consultation	The program consults with interested, affected and relevant parties.	6
Client feedback	The clients are given surveys or asked to provide feedback on the service.	6
At program beginning	Community involvement only during the inception of the program not continued.	1
Involve: cooperation and participation		
Community-led fundraising	The community raises money or contributes money to the program.	2
Training for community members	The program offers training to community members to provide animal healthcare themselves, for example lay vaccinator training.	2
Housed by the community	The program members are housed, fed, offered room and board by the community.	1
Collaborate: partnership in each aspect of project		
Invitation	The organization is invited to the community to provide services by the community.	4
Community employment/ volunteering	Individuals in the community itself are involved in assisting with organizing, planning, promoting, running the clinic.	8
Community developed staff training	The community has developed training for staff of the program including trauma-informed, cultural safety training.	1
Partnership with other community services	The program partners with social or other community service programs.	16
Shared Leadership: strong partnership structures; community makes final decisions		
Partnership with community leadership	The program develops a partnership with community leadership.	8
Community defined intervention goals	The goals of the program are chosen and defined by the community itself.	1
Working toward community independence	The program is aiming to help the community run the program themselves.	1
Other: topics not encapsulated by above 5-point spectrum		
Declining community involvement	Answers related to noticing or experiencing a decline in community involvement in the organization or service over time.	1
Need more community engagement	Answers related to a lack of community engagement and/or the need for more community engagement.	10
Unspecific	Answers that are unspecific in the community’s exact involvement or contribution to the program.	6
Unsure	Answers relating to being unsure about the question or how the community is involved.	2

5-point spectrum of community engagement drawn from ‘Principles of Veterinary Community Engagement’ (66).

‘**Consultation**’ involves a bi-directional flow of information, and some organizations noted that they seek feedback from their clients (*n* = 6) or the communities they work with (*n* = 6): “*We do seek their input in our strategic planning*” (NP40); “*Rightsholder engagement*” (MG2); “*We routinely reach out to our clients for feedback and comments regarding our services, what they need and evaluate how we can improve*” (NP26).

Other organizations made statements suggesting that they ‘**Involve**’ the community, for instance through training for community members (*n* = 2), community-led fundraising (*n* = 2), or the community providing room and board during the clinics (*n* = 1): “*Community-led fundraising for our programs that go directly to benefiting their members*” (NP11); “*We offer training of local vaccinators*” (ED3); “*We are housed, and fed by the community*” (NP17).

‘**Collaboration**’ involves a partnership throughout service planning and provision, and includes elements like being invited by the community (n = 4), providing employment or volunteer opportunities for community members (*n* = 8), community-developed training for organization staff (*n* = 1), and partnership with other community service programs (*n* = 16): “*Provision of all services began at the request of and in collaboration with all of the communities*” (ED4); “*Our Trauma-Informed & Culturally Safe training program was developed by a group which included Indigenous knowledge, and those currently or previously unhoused*” (NP13); “*require the communities to be stakeholders - to appoint liaisons who can help with organizing, promoting the clinic and providing some of the needs for the team*” (ED1).

Finally, ‘**Shared leadership**’ was demonstrated through participating organizations’ statements indicating a partnership with community leadership (*n* = 8), community-defined intervention goals (*n* = 1), and the goal of working toward community independence (*n* = 1): “*we always partner with local community governments or First Nations governments to bring accessible veterinary care to communities*” (MG1); “*Our role is to support a community in its journey toward a locally run management plan*” (NP4). The type of community involvement tended to vary with the community served. Indigenous Nations and very rural, remote, or geographically isolated communities tended to be more involved in organizational decisions than urban, low-income, unhoused, or precariously housed communities.

#### 3.3.3 Program impact and evaluation

Organizations reported various metrics to assess program impact, including: service availability, organizational input and effort, service utilization, animal health outcomes, and client perceptions of access to care (see Table 5 for Codebook with definitions and frequencies). The most common measure of program impact involved quantitative metrics of the numbers of services provided (*n* = 25): “*We just keep track of how many [animals] we were able to spay/neuter*” (NP8); “*Just numbers of medical and surgical appointments*” (ED2); “*Number of animals seen, number of repeat animals seen*” (ED5); “*how many pets supported in a year, how many guardians supported in a year*” (NP13). Another common measure involved client feedback (*n* = 7): “*We have distributed post clinic surveys (exit surveys) in past to gauge whether or not the communities felt our visit was a success*” (ED1); “*Feedback surveys including how services were accessed (transportation barriers), percentage waived fees, client experience with the service*” (NP10).

**Table 5:** Qualitative codebook for metrics used to measure impact of services including codes, sub-codes, definitions and frequencies reported in qualitative responses by 97 animal healthcare providing organizations in Canada who completed the organizational survey between December 5, 2023-February 20, 2024.

Codes and sub-codes	Definition	# of references
Service availability: Metrics related to the quantity and types of services available		
Service eligibility	Metrics related to amount of eligibility for services.	1
Service utilization: Metrics related to the communities’ actual utilization of the available services		
Service use trends	Metrics related to trends in client and patient use of services and the services provided over a period.	7
Animal census	Metrics related to conducting animal census in the community.	1
Animal demographics	Metrics related to animal demographics including numbers of pets	3
Client demographics	Metrics related to client demographics including	2
Health outcomes: Metrics related to changes in animal health before and after the intervention		
Individual health trends	Metrics related to trends in individual pets including their health history, physical exam, and changes in health outcomes.	3
Population health trends	Metrics related to population level trends in health in general or specific health outcomes that are of concern or are a focus of the service.	7
Effort: Metrics related to the immediate result of the intervention including services provided, clients and patients treated		
Services provided	Metrics related to number of, and type of services provided, or number/type of patients and clients provided with services.	25
Service provider perspectives	Metrics related to service provider perspectives on services provided, impact of services or experiences providing service.	2
Money raised	Metrics related to money raised by the clinic or services provided.	2
Input: Metrics related to the time and resources put into implementing the intervention		
Cost of services	Metrics related to the cost of services, total cost spent per pet or the cost of a program in total.	5
Staff resource input	Metrics related to the time and resources put into the intervention by staff, including staff training.	2
Perceived access: Metrics related to the community’s perspective, engagement, satisfaction and use of the intervention		
Client feedback	Metrics related to client feedback including experiences accessing the service measured through casual conversation, surveys, interviews, focus groups.	7
Community feedback	Metrics related to general community feedback including clients and non-clients through surveys, interviews.	2
Social media feedback	Metrics related to social media response, engagement, statistics, analytics and reach.	3
Evaluation in general: Answers related to evaluation of interventions including reasons to evaluate, type of evaluation and who is evaluating		
Metrics are difficult	Any responses related to trying to collect metrics but meeting great difficulty or not collecting metrics because it is difficult.	1
Formal research	Metrics associated with an academic research study to investigate the impact of a service or program.	1
Organizational review	Evaluation conducted by the organization themselves including board review.	1
Partner organization	Metrics are collected by or for a partner organization.	2
Reasons for metric collection	Answers relating to the reasons for collecting metrics or evaluating an intervention.	3

Other organizations used social media or website engagements (*n* = 3): “*Although biased, we also have a social media presence and the majority of the response is positive, but perhaps we are not capturing the negative*” (ED1); “*Facebook statistics and google analytics*” (MG3). Organizations also tracked financial measures including the amount of funds raised (*n* = 2) and the cost of services (*n* = 5): “*average dollar amount spent per pet (which is tied to the complexity of the cases we are taking on)*” (NP13); “*monthly/quarterly/annual cost*” (NP14); “*how much funding we raised*” (FP10).

Some organizations tracked individual animal health trends (*n* = 3), while others tracked population health trends (*n* = 7): “*pet history*” (NP7); “*tracking animals/clients over time to determine numbers of repeat animals and owners*” (NP20); “*medical records are used for chart reviews to understand change in animal health and welfare metrics over time (including age, BCS [body condition score], breed, sterilization status, vaccination status)*” (NP20); “*reduction in animal issues, reduction in stray animals*” (ED5). Some organizations noted tracking trends for particular health measures, such as tick-borne illnesses and heartworm, and animal (*n* = 3) as well as client (*n* = 2) demographics. Service use trends were also tracked (*n* = 7): “*booking capacity rate*” (FP14); “*Reduction in intake of targeted locations. Reduction in overall intake and owner surrender intake*” (NP26); “*[number of] new clients per month, [number of] new patients per month*” (MG4).

In terms of how gathered data were used, responses indicated that program monitoring or community feedback results were employed for the purposes of organizational review, to ensure objectives are being met, to formally publish or share knowledge, or to share with regulatory bodies as part of licensing requirements. Others noted that metrics were not collected or were challenging to collect: “*Not specifically*” (NP33); “*We work with organizations that collect metrics, but we do not collect metrics ourselves*” (NP44); “*We try but it is difficult*” (NP18).

#### 3.3.4 Other

One additional topic from the open-ended survey responses was a desire from respondents to do more, while acknowledging limitations of time and resources to do so: “*We would love to initiate programs to assist our community, however, we have very limited resources*” (NP3); “*we are very small and wish we were able to do more*” (FP9); “*I would like our organization to be able to offer more services, but unfortunately we are small*” (NP31); “*We need collaboration with similar groups and assistance from groups that have large financial means*” (NP44).

## 4 Discussion

A qualitative content analysis of specific barriers mentioned within survey responses strongly reflect barriers identified in other research, indicating that participating organizations seem to understand the main affordability, accessibility, availability, client situation and client-service provider relationship related barriers to care reported generally by clients in the animal healthcare literature (2–4, 7, 8, 10–12, 42–50). For instance, cost of care and appointment availability were key barriers noted by clients in a recent Canadian survey (2) and were also key topics identified by service providers within this study. More information is needed on the prominent barriers present for individuals and/or communities in Canada and whether local organizations are aware of context specific barriers and needs. Similarly, information from service-providers regarding their catchment areas (areas of service provision) reflects results from previous mapping of veterinary care deserts in Canada (39), namely large geographic areas in the Northern provinces and the east coast that seem to be underserved or have less availability to accessible care. Over 90% of organizations participating in this survey offered services in four provinces: Ontario, British Columbia, Alberta, and Quebec. Thus, either our recruitment methodology favored these regions and was not as successful at recruiting participants from eastern Canada, the prairies, and the Territories, or these regions may have fewer organizations providing veterinary care or working to mitigate access to care barriers. A dearth of organizations reporting to offer services in the Territories, with those who do primarily operating pop-up clinics and not year-round brick-and-mortar veterinary clinics, also aligns with existing knowledge of this substantial care desert (5, 6). Future research could target these areas in particular to understand in a more comprehensive fashion the extent of veterinary care deserts and strategies to improve the accessibility of veterinary care.

In terms of how organizations across the country are attempting to address barriers to accessing animal healthcare in underserved communities, over 90% of organizations mobilized tools related to service affordability or geographical access. Cultural and disability-related barriers were each mitigated by less than two-thirds of organizations. Organizations used an average of between 2 and 3 tools to mitigate financial and geographic barriers indicating that one tool is likely insufficient to increase access for diverse clients. The most common tools to mitigate financial barriers were offering free and low-cost services with most organizations working with low-income communities and almost half of organizations working directly with unhoused individuals. Only 15% of organizations surveyed offered payment plans without credit checks, which has been noted as an important tool to increase access by 14.5 times compared with providing full subsidies to fewer clients (67) and reduce the “pain of payment” (68).

Half of the organizations worked with rural communities and a third with northern communities who likely struggle with geographic accessibility. One of the most common tools used to mitigate geographic barriers was pop-up or periodic clinics while only 7% of organizations operate a permanent clinic in a rural, remote, northern community or Indigenous Nation. This indicates that even for those geographically underserved communities who have access to the services provided by organizations in this study, access is likely not regular. Telehealth services were only used by a third of organizations in this study despite gaining popularity during the COVID-19 pandemic with one study reporting a rise from 12 to 38% use by clinics between March–June 2020 (69). Telemedicine has been suggested to reduce cat stress and negative responses (70), increase accessibility of care compared to clinic visits (70–72), and increase clinic efficiency (73). However, some practitioners call into question the safety of telemedicine for new clients or new diagnoses without an in-person establishment of a veterinary-client-patient-relationship raising concerns about a lack of physical exam, missed or wrong diagnoses, technological constraints, and lack of diagnostic tools (71, 74, 75). Open-ended survey responses demonstrated a large range of strategies employed by organizations to target financial and geographic accessibility, showing a diversity of creative approaches and perhaps a need for further research on which tools are most impactful for individual community access.

Tools to mitigate cultural and disability-related barriers were less prevalent, with the average number of tools used by organizations to mitigate these barriers being less than one. Fewer than half of organizations trained staff on cultural safety, and less than 20% of organizations reported having diverse staff reflective of their clientele or offering services in multiple languages. Fewer than half of surveyed organizations had a physically accessible clinic, or trained staff on providing accessible service provision, and only one organization had assistive technology. Increased awareness and use of tools to meet the needs of individuals with disabilities is necessary to increase access to care (46). Given that the most common tool used to address cultural and disability-related barriers is staff training, future research should explore what this training consists of, who is developing the content, and how and by whom it is delivered, as this is likely to influence its effectiveness (76). The limitations of the current Canadian veterinary landscape with regard to cultural and disability related accessibility is an important area of focus to increase access to services, especially given the linkages between factors like health, socio-economic status, ethnicity, and disability (77–79). Relatedly, less than 40% of organizations used at least one tool from each of the four categories of barriers. The intersecting nature of access barriers makes clear the importance of taking a holistic approach that targets multiple axes of inequities (80, 81). Due to limited organizational resources and the availability of wide-ranging tools, future research should investigate the impact of individual tools used to increase access to care and the conditions that facilitate their usefulness in a community and organizational context.

Community involvement in programs ranged from simply accessing the service when it was available (outreach) to giving occasional feedback on their experiences (consulting), being employed or volunteering in program provision (collaborating), and community leadership partnering on initiatives (sharing leadership). Quite a few organizations partnered with other community service programs, demonstrating a One Health model (10), though ‘Promoting One Health’ was ranked as the lowest priority by survey respondents. This could be because some organizations are using partnerships to increase the use of their own program which primarily prioritizes animal welfare, or because they are not familiar with the term ‘One Health’, and a definition was not provided within the survey. These results in the context of the broader literature suggests that communities vary in capacity, interest, and needs regarding community participation or that organizations need resources to deepen their engagement with the diverse communities they serve (66). Future research should investigate community engagement methods in diverse communities to understand the best partnership and capacity-organizing practices.

Program evaluation most often involved organizations collecting quantitative measures of service usage. A smaller number of organizations tracked metrics like population health trends or solicited community feedback through surveys. The collection of only quantitative measures of service use, although useful, will make organizations providing fewer types of services to more clients look more impactful than organizations providing more holistic, long-term support to fewer individuals, which is not necessarily accurate, particularly at the individual level or over longer periods of time. Organizations reported primarily collecting metrics for funders, internal review, or organizational decision-making, all of which could significantly inform the organizational direction and service provision. All of the metrics reported by organizations to assess program impact (service availability, organizational input and effort, service utilization, animal health outcomes, and client perceptions of access) are reflective of barriers faced by clients and therefore each can be useful for understanding program impact to a certain degree, but none tell the complete story (36). There is often a tension in healthcare literature between a desire for a universal, standard, equal service and the development of services based on local needs and priorities (36), and this tension makes it challenging for organizations to know what to prioritize or measure, particularly if funders have impact report requirements. Participant comments that organizations try, or would like to conduct evaluations, but that they are challenging might reflect this very tension. More qualitative data is needed from these organizations with respect to their organizational goals and what access to care means, how and why organizations have developed these programs, what exactly is challenging about evaluation, and what types of support is needed to increase evaluation and community engagement capacity. Additionally, the low prevalence of long-term impact evaluation on health and community voiced needs by programs in Canada might indicate a lack of long-term service provision that is centered on community needs. Alternatively, it could indicate a lack of evaluation capacity in organizations who are providing sustained, relevant community care specifically with regard to community-engaged and continuous program evaluation and adjustment. In reality, these results are likely an indicator of both, and future research should seek to understand qualitatively how program evaluation plays a role in service development, improvement, and impact, what barriers restrict program evaluation, and how service providers would evaluate their program if given the resources and support to do so. These results highlight an area for potential capacity organizing resource sharing and collaboration between researchers, communities, organizations, and funders to make metric collection and program evaluation more accessible. Future research should collaborate with funders, organizations, and community members to create flexible best practices, guiding methodology and resources for program evaluation and impact measurement that is accessible and adds minimal administrative burden to organization staff, meaningful in relation to the needs of the target community, and useful for funders and decision makers to meet their goals (35, 82).

## 5 Limitations

This study had several limitations that might impact the generalizability or completeness of the data set with respect to Canadian animal healthcare organizations’ perspectives and practices. Selection bias is likely present due to the nature of a cross-sectional survey design and the use of convenience and snowball sampling (83). Participants might be statistically different (e.g., more interested or invested in access to care, have more time to participate in surveys, have more access and/or presence on social media, have more involvement with organizations who distributed our survey) than non-participants which would result in prevalence measurements of service availability, tool use or evaluation, and community involvement that might not be representative of the general population of organizations. If the population of organizations who participated were more likely to be more invested in the topics of the survey, this bias could lead to our prevalence estimates being higher than that of the general population. Additionally, this selection bias will impact the saturation of our qualitative data and therefore our content analysis might be some missing perspectives. In attempt to mitigate this, the survey was distributed as broadly as possible through online data mining, social media, and prominent regulatory bodies and national organizations. Additionally, the findings of the present study are discussed in the context of the population that participated and is intended to contribute to a growing understanding of the landscape of access to care in Canada. Future research could build on our database of organizations by targeting smaller geographic areas one at a time and using more diverse data collection methods including door-to-door survey delivery to attain more accurate prevalence estimates and qualitative perspectives for individual areas.

Recall and social desirability bias might also be present because participants were asked to self-report via online survey (83). Participants have the possibility of being incorrect in their recollection of their organization’s use of tools and metrics and therefore incorrectly answer questions. Participants might not want to answer certain questions truthfully due to fear or judgment or a desire to answer the questions in a way that helps the researchers with the objectives of the study. These biases were minimized by having the survey be anonymous, writing questions in a neutral non-leading way, and not sharing specific objectives of the study with participants. This bias was also addressed by limiting participation to those who felt confident to speak on behalf of their organization which might alternatively have resulted in missing organizational voices who do not have an individual willing or able to speak on their behalf.

Lastly, the options presented in multiple choice questions in the survey were written to include any possible answer that participants had yet these options still might force organizations to answer in ways that do not quite match how they feel. To mitigate this, survey questions were piloted with seven researchers and organizational leaders to ensure options were exhaustive and applicable. Additionally, “other” categories were available for all multiple-choice questions and mixed methods were used to allow participants room to expand in a qualitative manner.

## 6 Conclusion

This paper sought to use online data mining and a cross-sectional, mixed-methods organizational survey to determine how organizations across Canada are attempting to address barriers to accessing animal healthcare in underserved communities, and how program outcomes are being measured and evaluated. A total of 97 organizations responded to this survey, with a mix of nonprofit groups, for-profit clinics, and education institutions. Responses demonstrate that organizations mitigated access to veterinary care barriers primarily along financial and geographical lines, and to a lesser extent with tools targeting cultural or disability-related barriers. A large diversity of tools is mobilized by organizations, with some (no-cost or low-cost services, pop-up clinics) being more prevalent than others (payment plans, telehealth, services in multiple languages, physically accessible clinic).

Access to care can be hindered by many intersecting and wide-ranging factors, and it only takes one barrier to restrict an individual’s access to animal healthcare. Only 38% of organizations used at least one tool from each of the four categories of barriers, highlighting the importance of building capacity around addressing multiple intersecting barriers. Some organizations reported wishing that they could do more, but resources were limited, and organizations had funding limitations that were a barrier to providing more care. This corroborates the Canadian Veterinary Medical Association’s recent position statement that the need for veterinary care is significantly greater than the care available (84).

This research provides a characterization of the work being done within the private sector and by NGOs to address access to veterinary care barriers within Canada. It provides a necessary understanding of existing organizational efforts, impact evaluation challenges, and diverse perspectives on how communities are being partnered with. This sets the groundwork for evidence-based solutions to addressing animal healthcare service gaps and strategies to mitigate them most effectively through strong community partnership and community-engaged program evaluation.

## Data Availability

The raw data supporting the conclusions of this article will be made available by the authors, without undue reservation.
