# Supplementary material for: Access to veterinary care in Canada: a cross-sectional survey of animal healthcare organizations and interventions
Source: Front Vet Sci. 2025 May 30;12:1581316. doi: 10.3389/fvets.2025.1581316 (PMC12162722; doi:10.3389/fvets.2025.1581316)
Supplement: Supplementary file 1 [file Data_Sheet_1.pdf]

E-Survey: **Canadian Animal Healthcare Organizations Increasing Access to Veterinary Care**

1. What is your organization's **name**?
   - Open text box

2. Which of the following **most** applies to your organization?
   - For profit veterinary clinic
   - Not-for-profit organization, rescue, shelter, or clinic
   - Educational institution offering services in conjunction with student training
   - Other (please specify)

3. Which **species** do you offer animal healthcare services for? (Please select all that apply)
   - Dogs
   - Cats
   - Small animals (e.g., rabbits, guinea pigs, rats, mice)
   - Large/agricultural animals (e.g., horses, cows, pigs)
   - Exotic (e.g., birds, reptiles)

4. Within your organization, **who** provides these services to animals (check all that apply)?
   - Veterinarians
   - Veterinary technicians
   - Non-veterinary/technician employees or volunteers

5. In the context of your organization's effort to reduce barriers to accessing animal healthcare, please **rank** the importance of each of the following goals (with 1 being the least important and 6 being the most important – if you do not have an additional goal in the 'Other' category, leave it blank and rank it 1).
   - Improve animal welfare
   - Control animal populations
   - Help people whose pets are important to them
   - Prevent zoonotic disease
   - Promote One Health
   - Other (please specify)

6. What does your organization consider the **main barriers** to accessing animal healthcare?

7. Does your organization do any of the following to improve **financial access** to animal healthcare? (Please select all that apply)
   - **Free**/no-cost animal healthcare services (e.g., vaccines, spay/neuter)
   - **Low-cost**/subsidized prices for animal healthcare services (below the average cost of similar services in the community)
   - **Payment plans**/pay in installments for animal healthcare services that **do not** require credit checks
   - **Payment plans**/pay in installments for animal healthcare services that **do** require credit checks

- **Bundled services** at a reduced cost (e.g., sick-care packages to diagnose and treat sick animals, puppy/kitten packages or complementary services with the purchase of other services)
- **Spectrum of care** approach to treatment options to accommodate clients with financial constraints
- **Funding** available to assist pet care needs (e.g., grant funds, vouchers, discount programs)
- **Other** (please specify)

8. Does your organization do any of the following to improve **geographical access** to animal healthcare? (Please select all that apply)
   - **Mobile** unit of the clinic
   - **Pop-up/periodic clinics** in a different place than the 'brick and mortar' address (e.g., rural, remote, or Indigenous communities)
   - Primary clinic location in a **rural**, **remote**, or **Indigenous** community
   - **Telehealth** services
   - **Extended hours** of operation (outside of 9-5pm Monday-Friday)
   - **Transportation programs** or assistance to the clinic
   - Provision of **items** to assist with pet transportation (e.g., carriers)
   - **Other** (please specify)

9. Does your organization do any of the following to improve **cultural safety** and underserved community access to animal healthcare? (Please select all that apply)
   - Staff cultural safety **education/training**
   - **Diverse staff** (specifically equity seeking groups, &/or diversity reflective of your clientele)
   - Offer services in **languages** other than English (in dominant English-speaking areas) or French (in dominant French speaking areas)
   - **Other** (please specify)

10. Does your organization do any of the following to improve **access** to animal healthcare for **individuals with disabilities**? (Please select all that apply)
    - Staff **education/training** on equity and inclusion of clients with disabilities
    - **Physically accessible** clinic
    - **American Sign Language** interpretation
    - **Assistive technology** (any technological modality used to assist a person with a disability e.g., teletype device or telecommunications device for the deaf)
    - **Other** (please specify)

11. If applicable, please **describe** how your organization mitigates financial, geographic, cultural or disability related **access** to veterinary services.

12. What are your organization's **catchment area(s)**? (i.e., what are your places of operation and offering services if different from your 'brick and mortar' address – satellite clinics, popup clinics, etc.)

13. Which **underserved communities** specifically do you work with? (Please select all that apply)
    - Low-income pet guardians
    - Unhoused/homeless pet guardians
    - Rural communities
    - Northern communities
    - Indigenous communities
    - Other (please specify)

14. Relating to your answer from Question 13, please tell us about these **communities' involvement** in the creation, improvement, or evaluation of your programs.

15. Do you collect any **metrics** to **evaluate** the success of your program in mitigating barriers to access animal healthcare? If so, which ones?

16. Is there **anything else** you would like to share about your organization, access to animal healthcare, or the survey?
